# Health technology assessment for the acute and preventive treatment of migraine: A position statement of the International Headache Society

**DOI:** 10.1177/0333102421989247

**Published:** 2021-01-20

**Authors:** Hans Christoph Diener, Messoud Ashina, Isabelle Durand-Zaleski, Tobias Kurth, Michel Lantéri-Minet, Richard B Lipton, Daniel A Ollendorf, Patricia Pozo-Rosich, Cristina Tassorelli, Gisela Terwindt

**Affiliations:** 1 Institute for Medical Informatics, Biometry and Epidemiology, University Duisburg-Essen, Essen, Berlin, Germany; 2 Danish Headache Center, Department of Neurology, Rigshospitalet Glostrup, Faculty of Health and Medical Sciences, University of Copenhagen, Glostrup, Denmark; 3 Université de Paris, CRESS, INSERM, INRA, URCEco, AP-HP, Hôpital de l’Hôtel Dieu, Paris, France; 4 Santé Publique Hôpital Henri Mondor, Créteil, France; 5 Institute of Public Health, Charité – Universitätsmedizin Berlin, Berlin, Germany; 6 Départment d'Evaluation et Traitement de la Douleur, CHU de Nice, FHU InovPain, Universite Cete Azur, Nice, France; 7 Albert Einstein College of Medicine, Bronx, NY, USA; 8 Value Measurement and Global Health Initiatives, Center for the Evaluation of Value and Risk in Health, Institute for Clinical Research and Health Policy Studies, 1867 Tufts Medical Center, Boston, MA, USA; 9 Headache Unit, Neurology Department, Vall d'Hebron University Hospital, Barcelona, Spain; 10 Headache Research Group, Vall d'Hebron Research Institute, Universitat Autònoma de Barcelona, Barcelona, Spain; 11 Headache Science Center, IRCCS Mondino Foundation, Pavia, Italy; 12 Department of Brain and Behavioral Sciences, University of Pavia, Pavia, Italy; 13 Department of Neurology, Leiden University Medical Center, Leiden, the Netherlands

**Keywords:** Health technology assessment (HTA), migraine, acute therapy, preventive treatment, International Headache Society (IHS), position statement

## Abstract

The Clinical Trials Subcommittee of the International Headache Society presents the first Health Technology Assessment for the Acute Treatment of Migraine Attacks and Prevention of Migraine. Health technology assessments are systematic evaluations of the properties, effects, and consequences of healthcare technologies; this position statement is designed to inform decision makers about access to and reimbursement for medications and devices for the acute and preventive treatment of migraine. This position statement extends beyond the already available guidelines on randomized controlled trials for migraine to incorporate real-world evidence and a synthetic approach for considering multiple data sources and modelling methods when assessing the value of migraine treatments.

## Introduction

According to the World Health Organization, a health technology is the application of scientific knowledge, which can take the form of pharmaceuticals, medical devices, and procedures, as well as management, communication, and information systems, to solving health problems (1). Health technology assessments (HTAs) are characterized as systematic evaluations of the properties, effects, and direct and indirect consequences of a healthcare technology that are intended to inform decision makers (1). Ideally, HTAs identify and analyse the clinical, patient-related, organizational, economic, ethical, and legal issues associated with new health technologies to facilitate the evidence-based allocation of healthcare resources (2).

Viewed from a global perspective, the current use of HTAs to evaluate new medications and devices for the treatment of migraine is characterized by a lack of standardization, with different rules and criteria, data requirements, and methods of assessing benefits, risks, and costs at the regional, national, and local levels. The profusion of policies and the generation of disparate data sets increases the complexity and costs associated with the design and conduct of clinical trials; neglects an opportunity to streamline the oversight process; and prevents the development of international databases that might be used to improve real-world outcomes and identify areas of clinical practice that may be amenable to cost reductions.

To facilitate the development of an evidence base for migraine that clarifies the effects of new technologies for acute and preventive treatments for regulators and payers who rely on HTAs for decision making, the Clinical Trials Subcommittee of the International Headache Society presents the first Health Technology Assessment for the Acute Treatment of Migraine Attacks and Prevention of Migraine: A Position Statement of the International Headache Society.

## Objectives

The objectives of this position statement are to recommend global standards for the collection and analyses of evidence pertaining to new technologies (medications and devices) for the treatment of migraine and to facilitate HTAs that account for the distinctive nature of migraine and the heterogeneity of the affected population.

## Overall approach

The approach to HTAs for migraine should be evidence-based, systematic, reproducible, transparent to stakeholders, and comprehensive. While randomized controlled trials create high-grade, robust evidence, HTAs should also consider relevant real-world data, which can provide answers to questions that cannot be addressed in clinical trials, such as how health technologies for the acute and preventive treatment of migraine are introduced into clinical practice and used after they are approved or cleared. Taken together, the combination of trial and real-world data will better represent actual product value than trial data alone.

With respect to the collection of evidence that is relevant to HTA domains, data for assessment should have been published or submitted for publication at the time of evaluation. In circumstances where manufacturers’ confidential data would be useful for HTA assessments, HTA bodies should have processes in place to protect the confidentiality of such data in public reports or other documents.

## Health problem and current use of technology

Migraine is a chronic neurologic illness characterized by occasional attacks of moderate to severe headache lasting four to 72 h and associated with photophobia, phonophobia, and nausea (3). More than 1 billion people worldwide have migraine, and migraine is a leading cause of disability worldwide (4,5). Women are three times as likely as men to have migraine, and family and twin studies estimate a heritability of 42% (6,7). Age is also an important determinant of migraine risk and disability, as prevalence peaks in adults aged 35 to 39 years (8) – typically among the most productive years of life both personally and professionally. Because it is highly prevalent, migraine is the leading cause of disability among people aged 15 to 49 years (9); its negative effects have been shown to include substantial impairment in professional, academic, and social settings (10,11).

A range of technologies is available for the treatment of patients with migraine. Established, evidence-based options include analgesics, nonsteroidal anti-inflammatory drugs (NSAIDs), ergots, serotonin 5-HT_1B-1D_-agonists (triptans) for the acute treatment of migraine, and beta-blockers, flunarizine, anticonvulsants, amitriptyline and onabotulinumtoxinA for the preventive treatment of migraine (13). New technologies include small molecule calcitonin gene-related peptide (CGRP) antagonists (gepants) and 5-HT_1F_ agonists (ditans) for acute treatment and monoclonal antibodies (mAbs) to CGRP and its receptor for preventive treatment (13). New non-pharmacologic technologies for migraine include neuromodulatory devices, such as single-pulse transcranial magnetic stimulation, electrical trigeminal nerve stimulation, non-invasive vagus nerve stimulation, and remote electrical neuromodulation (13).

Despite the availability of established and new technologies, access to care varies considerably between and within countries, structured services are rare, and many people with migraine remain undiagnosed or receive suboptimal treatment (14). With limited resources, evidence-based policies are needed to address these challenges.

## Target population

The target population for established and new technologies comprises people who have migraine with aura, migraine without aura, or chronic migraine, as well as those who experience attacks of probable migraine, as specified in the latest edition of the International Classification of Headache Disorders (ICHD) (3). Because ICHD has been modified as the understanding of migraine has evolved (15–17), the most recent criteria should be applied when calculating outcomes in meta-analyses.

## Special populations

### Acute treatment of migraine

Patients with prior treatment failure, intolerance to prior treatment, contraindications to treatment, or for whom oral intake is not possible merit special consideration when assessing new technologies intended for the acute treatment of migraine.

#### Prior treatment failure

New technologies for the acute treatment of migraine can be compared with placebo in patients with a prior history of treatment failure. For HTAs, it is recommended that treatment failure be defined as an inadequate response at 2 h post dose on the endpoints of pain freedom or headache response (reduction of pain intensity from severe or moderate to mild or none) and freedom or response from the most bothersome symptom (MBS) associated with migraine (e.g. nausea, photophobia, or phonophobia) in at least three migraine attacks treated with an adequate dose and at the correct time of intake (not during aura) or stimulation. Treatment failure involving 5-HT1B/1D receptor agonists (triptans) should be defined as an inadequate response on the endpoints listed above to at least two different triptans alone or in combination with NSAIDs.

#### Intolerance to prior treatment

New technologies for the acute treatment of migraine can be compared with placebo in patients who have a history of intolerance to prior treatment due to adverse events (AEs). For patients reporting mild intolerance to triptans, a history of poor tolerability to at least two different medications in the class should be documented. In case of moderate or severe intolerance to a drug, no further exposure for the same class of drugs is required.

#### Contraindications to treatment

New drugs for the acute treatment of migraine can be compared with placebo in patients who have contraindications to established therapies, such as a history of coronary artery disease with nonsteroidal anti-inflammatory drugs or uncontrolled severe hypertension, ischemic heart disease, stroke, or peripheral arterial disease with triptans.

#### Oral intake is not possible

New technologies for the acute treatment of migraine can be compared with placebo in patients who are unable to use oral medication because of early vomiting during attacks or difficulties swallowing oral tablets or capsules. Non-oral acute treatments may involve alternative formulations of an orally-administered medication (e.g. parenteral, nasal spray, suppository) or a neuromodulatory device.

### Preventive treatment of migraine

Patients with prior treatment failure, intolerance to prior treatment, contraindications to treatment, or with chronic migraine and medication overuse merit special consideration when evaluating technologies intended for the preventive treatment of migraine.

#### Prior treatment failure

New technologies for the preventive treatment of migraine can be compared with placebo in patients in whom two classes of preventive treatments, each taken regularly in adequate doses for at least 3 months, were considered to have failed.

#### Intolerance to prior treatment

New technologies for the preventive treatment of migraine can be compared with placebo in patients who have terminated two or more classes of migraine preventive treatments due to AEs, with classes defined as medications or devices that have been approved or cleared for migraine or have demonstrated efficacy in two or more randomized controlled trials.

#### Contraindications to treatment

New technologies for the preventive treatment of migraine can be compared with placebo in patients in whom two or more classes of migraine preventive therapies are contraindicated. Valproate and related substances (valproic acid, sodium valproate, valproate semisodium, and valpromide) are contraindicated in pregnancy and in women of childbearing potential who are not using effective methods of contraception (18).

#### Chronic migraine and medication overuse

New technologies for the preventive treatment of migraine can be compared with placebo in patients with chronic migraine and medication overuse who failed preventive therapy with onabotulinumtoxinA, topiramate, and monoclonal antibodies (mAbs) against CGRP or its receptor; terminated treatment due to AEs; or had contraindications.

### Acute/preventive treatment

For both main types of migraine treatment, data from randomized controlled trials are usually lacking or very limited in women who are pregnant or breastfeeding, children, and adolescents. Prospective registries can provide information from women who become pregnant during preventive drug therapy or who take acute medication during pregnancy. Because most migraine trial populations are at least 70% female, males tend to be under-represented in results from randomized controlled trials. It is therefore recommended that assessments be weighted as necessary to account for these gaps in the migraine evidence base.

## Migraine frequency

Frequency is an important tool in migraine classification. It is the cardinal feature of chronic migraine, defined as at least 3 months with 15 or more MHDs, at least eight of which satisfy criteria for migraine (3), and establishes by exclusion a diagnosis of episodic migraine (patients with migraine who do not have chronic migraine are assumed to have episodic migraine). The term “high-frequency episodic migraine”, which describes patients with migraine who experience 10–14 MMDs (19) often with severe disability (20), can be helpful in understanding preventive treatment effects in those at the highest risk of progression to chronic migraine.

It is recommended that HTAs assess migraine frequency by counting days with migraine per month or days with headache per month, and the respective units of measure should be monthly migraine days (MMDs) and monthly headache days (MHDs). A migraine day is defined as any calendar day on which the patient had onset, continuation, or recurrence of a migraine headache. Any calendar day on which acute migraine-specific medication (i.e. triptan or ergotamine) is used is also a migraine day. Alternatively, a time period of 28 days can be used. For HTAs of technologies for the preventive treatment of migraine, MMDs should be preferred because they eliminate the need to account for acute medication use and attack interruption due to sleep (21). For assessments involving individuals with chronic migraine, MHDs are preferable to MMDs because the phenotype can change over time, and not all headaches fulfil diagnostic criteria for migraine (3).

The use of frequency in HTAs for migraine has limitations, as it does not account for headache pain intensity or migraine-related disability. For example, patients with relatively few MMDs whose migraine attacks are severe, long lasting, or incapacitating may benefit considerably from a course of preventive treatment, particularly if acute therapies are ineffective or inconsistently effective. Accordingly, HTAs of preventive treatments should utilize a comprehensive approach that incorporates the frequency, severity, and duration of migraine attacks, as well as any decrements in functional ability associated with them (22).

## Comparators

The developers of new health technologies typically conduct randomized trials to demonstrate efficacy using placebo as a control. For HTAs in migraine, it is recommended that new technologies be compared with an established treatment in the specific patient population of interest. If head-to-head trials comparing relevant interventions are not available at the time of assessment, HTAs should employ indirect or mixed-treatment comparisons (e.g. network meta-analyses) to estimate relative effectiveness, provided that the trials being analysed have comparable treatment periods, endpoints, assessments, and populations (23). Whenever required, statistical adjustments for potential confounders of treatment effects should be included. The HTA process should be fully inclusive of relevant stakeholders, particularly patients and/or patient groups (including public hearings by the HTA authorities)

### Acute treatment

Appropriate comparators for technologies intended for the acute treatment of migraine include the oral tablet formulations of sumatriptan 50 mg, sumatriptan 100 mg, rizatriptan 10 mg, or eletriptan 40 mg and 80 mg. For new technologies intended for patients who do not respond to triptans, do not tolerate them, or have cardiovascular contraindications that preclude their use, appropriate comparators include a different class of medications (e.g. NSAIDs), usual care, or placebo. With usual care as the comparator, real-world data on headache patterns and treatments are required, and patients who have a history of failure with currently available treatments should be analysed. The main challenge with using placebo as a control is that results may lack external validity; placebo is not given as a treatment for migraine in clinical practice.

### Preventive treatment

Comparators that may be used for efficacy trials technologies intended for the preventive treatment of migraine include propranolol 80 mg to 160 mg, metoprolol 100 mg, topiramate 50 mg to 100 mg, flunarizine 5 mg/day to 10 mg/day, amitriptyline 25 mg to 50 mg, and mAbs against CGRP or the CGRP receptor (e.g. monthly erenumab 70 mg to 140 mg, fremanezumab 225 mg, or galcanezumab 120 mg and quarterly eptinezumab 100 mg). For populations that include patients with chronic migraine, appropriate comparators include onabotulinumtoxinA 155 IU to 195 IU; topiramate 50 mg to 100 mg; monthly erenumab 70 mg to 140 mg, fremanezumab 225 mg, or galcanezumab 120 mg; and quarterly eptinezumab 100 mg.

#### Acute/preventive treatment

Comparisons involving new technologies for the acute and preventive treatment of migraine require that HTAs consider multiple dimensions (e.g. clinical, patient-reported, economic), but in patients with migraine it is especially important that assessments incorporate individual and societal needs. Since most current drugs for the treatment of migraine are available in generic form, new drugs will rarely be superior to them in cost-benefit analyses. Moreover, in migraine prevention trials there seems to be a biological threshold of 50% for a ≥50% reduction in attack frequency (24); indirect comparisons between different preventive drugs show a success rate between 20% and 45% for the 50% responder endpoint (24,^25^). Therefore, new technologies are unlikely to outperform established technologies if assessors focus on findings within the clinical domain.

To facilitate the development of HTAs that account for methodologic issues in migraine clinical trials, allow for the individualization of treatment effects in the target population, and maximize the cost-effective allocation of resources, it is recommended that HTAs emphasize patient-reported outcomes, specifically, treatment-related improvements in quality of life and productivity (absenteeism and presenteeism), which represent a large percentage of the total costs associated with migraine (26). It is also recommended that HTAs determine the relative costs and benefits associated with different approaches to the treatment of migraine. In particular, requiring that patients fail two or more classes of existing acute or preventive technologies before CGRP-targeted technologies can be initiated may restrict access to care by undervaluing improvements in safety and tolerability, quality of life, and productivity versus existing technologies (27).

#### Outcomes

The endpoints and/or outcomes used in HTAs can differ from the primary endpoint used in clinical trials, but they must be relevant to the multiple constituencies that contribute to the overall value of an intervention, including clinicians, patients, caregivers/partners, healthcare systems/payers, and employers/societies (Table 1).

**Table 1.** Endpoints for the evaluation of technologies for the acute and preventive treatment of migraine and their relevance for different stakeholders.

	Clinicians	Patients	Caregivers and partners	Health system and payers	Employers and society
Acute treatment					
Pain freedom	×	×	×	×	×
Freedom or relief from the MBS	×	×			
Functional disability	×	×		×	×
Preventive treatment					
Reduced use of acute treatment(s)	×	×	×	×	×
Reduction in MMDs	×	×	×	×	×
Reduction in attack severity	×	×	×	×	×
Acute/preventive treatment					
Tolerability and adverse events	×	×	×	×	
Serious adverse events	×	×	×	×	
Cost of treatment					
Direct		×		×	×
Indirect		×		×	×
Adherence and persistence	×	×			×
Patient-reported outcomes					
Quality of life	×	×			×
Patient satisfaction/preference	×	×			
Reduction in absenteeism		×		×	
Reduction in presenteeism^†^		×		×	
Increase in household productivity	×	×	×	×	
Practice efficiency				×	×

MBS: most bothersome symptom; MMDs: monthly migraine days.

^†^Working while ill, resulting in reduced productivity.

Ideally, the outcomes data required for HTAs should have already been collected in randomized controlled trials conducted for approval by regulatory bodies and in long-term observational studies, post-approval studies, and prospective registries designed to collect data on efficacy, tolerability, safety, and patient-reported outcomes, and can be used to complete all domains of the European Network for Health Technology Assessment’s Core Model (28). Data collection for HTAs should only be initiated in the presence of a proper power calculation that considers the primary and the most important secondary outcomes and an estimated drop-out rate. For pain freedom at 2 h in acute treatment trials, power should be calculated to estimate the contrast of the absolute risk. For the mean reduction in MMDs in migraine prevention trials, power should be based on estimated difference in the absolute risk. Data relating to patient experience and preference, as well as properties of the product that facilitate adherence and persistence (e.g. patient satisfaction, tolerability) should also be considered, as it can be associated with more favourable outcomes in clinical practice. Unlike randomized controlled trials performed for regulatory approval, HTAs can analyse the intention-to-treat (ITT) or as-treated population.

### 

#### Efficacy

In principle, all outcomes in the acute or preventive treatment of migraine attacks are patient-reported outcomes and, therefore, subjective.

#### Acute treatment

Pain freedom and freedom from the MBS at 2 h (29): Pain is one of the most disabling symptoms of migraine attacks, but some patients report being even more bothered by nausea/vomiting or photophobia. For HTAs involving acute treatments, the primary endpoint should be pain freedom at 2 h after treatment or freedom or relief from the MBS at 2 h after treatment. These straightforward efficacy measures can be captured prospectively and over a long period via the use of headache diaries. Emerging observations suggest that for some patients the most bothersome symptom may include pain exacerbation with movement or cognitive effects of migraine.

Sustained pain freedom (at 24 or 48 h): Ideally, acute migraine treatment should abort migraine attacks. Because migraine attacks can last up to 72 h, and many acute treatments have a shorter half-life, it is important to record relapses of headache pain that occur after an initial (2-h) response to treatment.

Consistency of response: For patients with migraine, a reliable response across migraine attacks is an important benefit of treatment (30). Since many studies of acute treatments employ single-attack designs, it is important to consider the results of multiple randomized controlled trials in HTAs. Consistency can be measured at the population level or the within-person level. Population consistency is assessed by measuring the proportion of patients achieving an endpoint (2-h pain freedom, for example) for the first attack, the second attack, the third attack. Within person or intra-individual consistency is assessed by measuring the proportion of patients achieving an endpoint in a pre-specified proportion of attacks; examples include two of three, three of three, three of four or four of four attacks. Alternatively, within-individual consistency can be assessed using estimates of variability.

#### Preventive treatment

Reduction in MMDs or MHDs: The primary endpoint for HTAs involving preventive treatments should be the reduction in MMDs versus baseline over a time period of 3–6 months, which are relatively easy to define and capture. For those with chronic migraine, MMDs and MHDs are equivalent, as migraine features tend to become attenuated as the frequency of headache days increases. Since reduction in MMDs may be explained by the occurrence of fewer attacks, the reduction in attack frequency should be a secondary outcome measure in prevention trials.

Responder rate: The 50% response rate is defined as the proportion of participants who have a 50% or greater reduction in monthly migraine days. The 50% response rate is adequate for episodic migraine. For chronic migraine, a 30% rate is clinically relevant (31).

Other: Other endpoints that may be clinically relevant in the assessment of preventive treatments include reductions in the number of acute medications taken, days of acute medication intake, intensity of migraine attacks, and duration of migraine attacks. All these measures are sensitive to the attenuation of disease severity and can be easily monitored and quantified.

If feasible, prevention trials should be conducted separately for patients with episodic migraine (4–14 attacks per month) and those with chronic migraine (≥15 MHDs for >3 months with migrainous features on ≥8 days/month). Variance in the magnitude of the placebo effect for some secondary measures between episodic migraine and chronic migraine can complicate the interpretation of placebo-adjusted treatment effects.

#### Patient-reported

For analyses based on quality-adjusted life years (QALYs), generic quality of life instruments can be included as secondary endpoints in clinical studies, but these generic instruments may be less sensitive to the fluctuating impact of migraine on patients’ well-being. Alternatively, migraine-specific instruments can be included as secondary endpoints, and the data can be mapped to more general quality of life instruments to support the estimation of QALYs. The use of different instruments should be based on the strengths and limitations of each instrument, the context of the clinical study, and the context of subsequent HTA evaluation. Within the many instruments for patient-reported outcomes, the Headache Impact Test (HIT-6), the Migraine Disability Assessment (MIDAS) scale and the Migraine-Specific Quality of Life questionnaire (MSQ v2.1) are frequently used (32) and have a reasonable reliability. Some items used in patient-reported outcomes can be collected via migraine apps.

#### Headache Impact Test

The Headache Impact Test (HIT-6) (33), which has a 1-month recall period, is recommended for assessment of migraine-related impact or disability (34).

#### Migraine-Specific Quality of Life questionnaire

The Migraine-Specific Quality of Life questionnaire (MSQ version 2.1) is recommended to evaluate the change in quality of life related to episodic and chronic migraine (35). This measure has a global scale and three subscales. In the USA, the Role Function Physical subscale is considered valid by the Food and Drug Administration and is included on prescribing information for migraine treatments. (https://eprovide.mapi-trust.org/instruments/migraine-specific-quality-of-life-questionnaire).

#### Migraine Disability Assessment questionnaire

The Migraine Disability Assessment (MIDAS) questionnaire (36) was originally validated using a 3-month recall period, but 4-week recall forms have been developed and used in clinical trials (37). MIDAS can provide estimates of absenteeism and presenteeism (38). MIDAS score data can be reported as time lost due to migraine, which is calculated as absenteeism plus 50% of presenteeism.

#### Health-related quality of life and generic

Validated, disease-specific health-related quality of life (HRQOL), productivity, and generic instruments are recommended as secondary endpoints. For some of the instruments listed in this section, the between-group minimal important difference (MID) has been defined in migraine and used in clinical trials of treatments for episodic migraine (34,39).

Patient Global Impression of Change. The Patient Global Impression (PGI) scale is the patient-reported counterpart to the Clinical Global Impression (CGI) scale, and it can be used to determine if there has been a change (improvement or decline) in clinical status (PGI-C) by evaluating patients' beliefs about the efficacy of treatment. Available in the public domain (40), the PGI-C asks how patients are doing overall at pre-specified time points (e.g. 4, 8, or 12 weeks after baseline) compared with their pre-treatment baseline using a seven-point scale where 1 = very much improved and 7 = very much worse.

Functional Impairment Scale: The Functional Impairment Scale is a four-point scale that addresses functional status and intensity of impairment during daily activities (41,42). It can be used in conjunction with the four-point pain intensity scale and is usually completed on a daily basis and summarized over 4-week intervals.

Migraine Functional Impact Questionnaire: The Migraine Functional Impact Questionnaire (MFIQ) is a 26-item self-administered instrument that assesses the impact of migraine on physical functioning, usual activities, social functioning, and emotional functioning over the past 7 days (43).

Migraine Physical Function Impact Diary (MPFID). The MPFID is a 17-item daily diary designed to measure the impact of migraine on physical functioning (44). This instrument focuses on physical impairment and impact on everyday activities over the past 24 h, and it allows for evaluation of migraine’s effects on ictal days (when attacks occur) and interictal days (between attacks) (44). In the United States, the MPFID is considered valid by the Food and Drug Administration and is included on prescribing information for migraine treatments.

Work Productivity and Activity Impairment (WPAI) Questionnaire: Migraine is highly prevalent among working-age adults (8), and work-related productivity losses are an important component of the total migraine experience. These losses can be assessed with the Work Productivity and Activity Impairment (WPAI) questionnaire, which has a 7-day recall period (45). Created as a patient-reported quantitative assessment of absenteeism, presenteeism, and daily activity impairment attributable to general health, the WPAI has been validated in several conditions, including chronic pain and migraine (46,47).

#### Generic instruments

EuroQoL-5 Dimension Questionnaire: The EuroQoL-5 Dimension Questionnaire (EQ-5D) is a self-administered standardized measure of health status that captures a patient’s situation on a particular day (48). Although all levels of migraine pain have been associated with significantly reduced utility values (49), it is recommended that interpretation of EQ-5D scores in HTAs of technologies intended for the treatment of migraine distinguish headache days from headache-free days, as disutilities during migraine attacks are much greater than those reported when migraine is evaluated as a chronic health condition, with disutilities collected at random times during and between attacks (50). It is also recommended that EQ-5D scores be mapped to MMDs, as MMD reductions are likely to impact health state utility increments.

36-Item Short Form Health Survey: The 36-Item Short Form Health Survey (SF-36) is a generic instrument for the evaluation of quality of life (51) with a recall period of 4 weeks. Recommended uses for the SF-36 in migraine HTAs include comparing the effects of technologies for acute or preventive treatment of migraine on quality of life or comparing the benefits of a migraine technology with a technology used to treat another condition, such as asthma. In acute treatment trials, measurable benefits develop over 3–6 months (52).

### Tolerability and side effects

For the evaluation of the tolerability and safety of migraine technologies, it is recommended that HTAs collect all-cause discontinuations, discontinuations from AEs, serious AEs, and any AE reported by at least 5% of the treated population. With randomized controlled trials of technologies for the preventive treatment of migraine, data about the persistence of AEs over various time periods (3, 6, and 12 months) should also be collected.

### Surrogate

It is recommended that surrogate outcomes, such as biomarkers or results from imaging or electrophysiological studies, not be considered until validation of their surrogacy for clinical endpoints is provided.

### Treatment costs

Due to the complexity of assessing health technology usage in patients with migraine (i.e. a chronic condition with intermittent symptoms), it is recommended that HTAs of migraine technologies consider the direct and indirect costs of care, including trial results, characteristics of patients enrolled in the trial (e.g. episodic vs. chronic migraine), and the timing and dose of the technology. Although most national HTA bodies use a healthcare payer perspective that focuses on direct costs, migraine imposes a considerable burden on patients, families, and health systems. Attempts should be made to quantify the amount of work and leisure time lost, despite the fact that to date no consensus has been reached regarding the methodology for valuation, or the impact of this approach on the relevant threshold for decision-making. At a minimum, the contribution of direct and indirect costs to the burden of migraine, and the impact of health intervention on these costs should be measured and compared.

The objective of an economic evaluation is to raise awareness about the economic implications of various treatment options among payers and policy makers. For HTAs in migraine, it is recommended that data on healthcare resource utilization be drawn from trial-based collected data, claims data, prospective or retrospective patient-level data collection, or literature reviews (53,54). If the influence of a new treatment on direct and indirect costs needs to be assessed over a longer period of time than permitted by standard data collection (e.g. lifetime), modelling with decision trees, patient-level microsimulations, or Markov cohort models can be used as part of a broader effort to assess cost-effectiveness (55).

The use of Markov models requires specifying the characteristics of the patient sample whose costs are being modelled, selecting treatment groups, and a plan for estimating medical costs. The characteristics of the patient sample should include a distribution of MMDs and MHDs at baseline. Since models depend on assumptions about the nature of the distribution of MMDs or MHDs, and it is unrealistic to assume that migraine attacks are normally distributed, acceptable choices (with appropriate testing of model fit) include Poisson, zero-inflated negative binomial, and Beta distributions (56). Estimates of the effect of technology on that distribution, as well as the direct and indirect costs associated with an MMD or an MHD, should be made. The patient sample should resemble the target population for the new technology to make the model clinically relevant. Recommended treatment groups include a new technology versus placebo, a standard treatment, or usual care, depending upon the goals of the developer. The selection of treatment arms should include data on the effects of treatment from randomised trials, long-term extension studies, or real-world evidence. Plans for estimating direct and indirect medical costs should include changes in those costs associated with use of the technology.

#### 

##### 

###### Direct costs

The possible direct costs of migraine include medication for acute and preventive treatment; management of treatment related AEs; outpatient therapy; office visit(s) with general practitioners, non-neurologic specialists (internists, paediatricians, ob-gyn), neurologists, psychologists; telehealthcare visits or consultations; diagnostic evaluation and management of treatment-emergent side effects; home visits by the medical emergency service (available in some countries); emergency room visits; physical therapy; in-patient therapy; and management of comorbid conditions (57–61).

###### Indirect costs

Indirect costs arise from lost time in at least three domains: paid work; unpaid work (attending educational activities [i.e. school or university], household work, chores, caregiving, volunteer work); and family, social, and leisure activities. For each domain, indirect costs arise from missing activities (i.e. absenteeism) and from reduced effectiveness in a domain (i.e. presenteeism). In addition, illness may lead to long-term effects that are difficult to model, such as under-employment or unemployment, education, and career limitation (62). To ensure comprehensive assessments and accurate decision making, it is recommended that HTAs in migraine account for absence from work or school; presenteeism (reduced productivity during attacks at work or school), days of missed unpaid work, hours of lost or reduced productivity in unpaid work, restrictions on activities in the private sphere (e.g. caring for children or family members); and unemployment or early retirement.

Lost time in each HTA domain should be estimated and then monetized by quantifying its economic value. For technologies used for acute treatment, the usual approach to modelling assumes a distribution of migraine attacks and a distribution of benefits of acute treatment on the treated attacks; acute treatments generate direct cost savings by reducing utilization of technologies related to attacks. The indirect costs savings derive from allowing people to go to work (reducing absenteeism) or by reducing disability at work (reducing presenteeism), and they are calculated by multiplying hours of a missed activity by the economic value of the missed activity (e.g. for paid employment, the value per hour of lost work equals the salary per hour worked). While this strategy can be used to show how annual healthcare costs for patients with migraine increase with MMDs, the challenges in estimating value are more complex in the domains of unpaid work and family, social, and leisure activities.

## Validity

Randomized controlled trials top the hierarchy of evidence for the assessment of new health technologies due to their high internal validity and reduction of confounding. However, randomized controlled trials may have limited external validity with respect to the generalizability of results to a real-world setting. There are many issues affecting the external validity of a randomized controlled trial, including the setting, selection and characteristics of patients, similarities to/differences from routine clinical practice, outcome measures, follow-up, and AEs (63). The representativeness of a trial population is a particular problem because it is limited by patient inclusion/exclusion criteria, restricted sample size and exclusion of special populations (children and adolescents, elderly, pregnant and breastfeeding females, patients with comorbid conditions). To improve the external validity of randomized controlled trials, it is recommended that clinical trials in migraine be modified to include a more representative patient sample and/or a supplementation with real-world evidence (64).

Trial design modification is limited by the possibility of compromising internal validity. Beyond comparing characteristics of patients with those of patients in the clinical setting likely to be considered for drug or treatment, there is a need to develop methods to assess the generalizability to the target population. Among these statistical methods, reweighting available data from randomized controlled trials based on existing patient characteristic data from a target population is an exploratory approach to generalizing randomized controlled trials data to real-world patient populations (65). The advantage of reweighting is that it only requires observational baseline patient characteristic data, which are more likely than observational outcome data to be available at the time of market access and reimbursement regulatory decisions.

The supplementation with real-world evidence is possible using different sources, including patient surveys, patient registries, healthcare databases (including electronic health records), pharmacy and health insurance databases, social media and patient-powered research networks. All of these, however, suffer from selection biases that can affect valid inference. Using these sources, real-world evidence is usually provided by observational studies. However, real-world evidence is difficult to implement because it is rarely available for a new drug or treatment at the time of market access and reimbursement regulatory decisions. On the other hand, real-world evidence is collected in post-registration studies requested for the reassessment to increase the precision of modalities of use, the effectiveness and the tolerability and safety of a drug or treatment in a non-selected population over prolonged durations.

### Indirect comparisons

For HTAs in migraine, indirect comparisons between current and new technologies are warranted, and several techniques are available. The network meta-analysis methodology permits comparison of each therapy to a uniform comparator (e.g. placebo, standard of care) and/or to each other. Matched adjusted indirect comparisons permit the estimation of efficacy between published results and individual patient level data via estimation of the probability that a given patient might be enrolled in a trial (based on published entry criteria). These probabilities become “weights” that can be used to estimate adjusted efficacy and safety outcomes from individual patient data that are compared to the published data (66).

## Stopping rules and implementation

### Acute treatment

Medications and neuromodulatory devices used for the acute treatment of migraine are considered ineffective if they do not demonstrate a significant difference or non-inferiority from a comparator on the endpoints of pain freedom or headache response (pain intensity from severe or moderate to mild or no pain) 2 h after dosing (lack of response). After an initial response to acute treatment (i.e. at 2 h), headache pain of moderate or severe pain intensity may return within 24 to 48 h (i.e. relapse). Treated migraine attacks characterized by a lack of response at 2 h or relapse within 24 to 48 h after treatment may be regarded as treatment failure. However, before a decision is made to stop using a particular treatment, three attacks should be treated, and it should be confirmed that treatment was appropriately administered (e.g. adequate dosage and time of administration). Cessation of an acute treatment should be considered if lack of response or relapse occurs in more than one of the three attacks.

### Preventive treatment

Migraine has a highly variable expression over time, and its natural course typically includes spontaneous improvements and regressions (67). Therefore, preventive treatment might be paused after a certain time period to determine whether the post-treatment pattern of migraine attacks (frequency and severity) warrants continuation of the preventive therapy. There are, however, very few randomized studies investigating termination or continuation of migraine preventive therapy (68).

A response to preventive treatment of migraine can be assumed if patients experience i) a reduction in mean MMDs of at least 50% in patients with episodic migraine or at least 30% in patients with chronic migraine relative to the pre-treatment baseline (31) confirmed by diary documentation or healthcare provider attestation or ii) a clinically meaningful improvement on the Migraine Disability Assessment (≥5 points for baseline 11–20 or ≥30% for baseline >20) (69); Migraine Physical Function Impact Diary (≥5 points) (70); or Headache Impact Test (≥2.5–6 points) (71,72).

The recommended duration of treatment for the prevention of migraine attacks is at least 3 months, and efficacy evaluations should be based on comparisons of the baseline assessment (4 weeks prior to start) with data from the last 4 weeks of the 3-month (12-week) treatment period. The termination of preventive treatment should be recommended to patients if a response is not achieved after 3 months for most migraine preventive drugs and 6 months for onabotulinumtoxinA and CGRP mAbs.

When treatment is determined to be effective and well tolerated for the prevention of migraine attacks, it should be continued for at least 12 months. After 12 months, medication can be paused for 4–8 weeks to evaluate whether treatment is still necessary, as nearly half of patients may no longer require preventive drug therapy after responding to long-term preventive treatment (e.g. MMDs reduced by 50%). If the frequency of MHDs or MHDs increases during the hiatus, the treating physician should recommend another 12-month cycle of preventive medication, with the decision to proceed made jointly by the patient and the treating physician.

## Economic evaluation, modelling of benefits, QALYs, and incremental cost-effectiveness ratios

Most HTA bodies employ a health-system perspective in economic modelling. The rationale varies by setting, but considerations include the difficulty in attributing certain societal benefits (e.g. educational benefits, disability expenses) to a single intervention, the unintended consequences of favouring healthy, working individuals over individuals in more marginal areas of society in a societal perspective, and the inability (in some settings) of health system budgets to address societal concerns. Still, there is general empathy for the effects of interventions on productivity, caregiver burden, and other societal benefits, effects that may be particularly important for migraine treatments. The Second Panel on Cost-Effectiveness in Health and Medicine recommends that both perspectives be used in every cost-effectiveness analysis to provide a set of boundaries for results and identify whether perspective makes a material difference in findings (73).

Models estimate the cost of treating a cohort of patients with migraine for a clinically relevant time period. Although the time frame of the model is somewhat constrained by the availability of data on long-term treatment effects, models can evaluate time periods from 3 months to several years/lifetime. For HTAs in migraine, the time frame should be clinically plausible and reflective of the reference case (the standard approach for modelling, as defined by a given HTA body). For the evaluation of the treatment of migraine attacks, a 6–12-month time period is recommended. For migraine prevention, a 3-year period is reasonable. Models should make realistic assumptions about continuing or discontinuing therapy based upon response; patients who do not respond to treatment are unlikely to continue for extended periods of time.

Assumptions about weaning or discontinuation should be built into the models and informed by evidence from clinical trials or real-world data. Treatment guidelines in many countries recommend a pause in preventive treatment after 12 months to determine if migraine prevention is still necessary (74–78). Models depend on assumptions about the nature of the distribution of MMDs or MHDs. Since it is improbable that migraine attacks are normally distributed, appropriate choices for use in modelling MMDs include Poisson, zero-inflated negative binomial, and Beta distributions (56). With appropriate testing of model fit, all are acceptable.

Models should specify a primary outcome variable. The recommended primary outcome variables include incremental cost-effectiveness ratios (cost per quality- or disability-adjusted life year gained). From a societal perspective, models should include both direct and indirect costs. Models developed from a payer perspective exclude indirect cost.

Sensitivity analyses should explore incremental cost-effectiveness ratios in clinically relevant subgroups defined by diagnosis (episodic vs. chronic migraine), monthly migraine day frequency, baseline levels of disability (productivity losses) or health-related quality of life. In addition to the deterministic sensitivity analyses, probabilistic sensitivity analyses should explore the impact of parameter variation on the uncertainty relevant to the joint distribution of costs and outcomes. The structural uncertainty of the model chosen is best explored with scenario analysis on: The choice of the type of model, the selection of health states, patterns of intervention, alternative methods for extrapolating health outcomes after the end of the observation period, and the cycle length (79).

Even with a societal perspective, HTAs should not rely solely on a QALY-based approach for determining value, as QALYs do not capture the full value of an intervention due to immature methods for estimating certain domains, insensitivity of utility instruments to specific patient preferences, and benefits not measured in clinical studies. Rather, QALYs should serve as a starting point that can be augmented by other elements such as patient preferences and risk tolerance, as well as by novel elements of value. HTA bodies should integrate contextual elements and additional benefits into their discussions and deliberations, and possibly consider adjusting their cost-effectiveness thresholds to reflect these elements if their contribution can be quantified. Examples include the availability of multiple treatments, the severity of conditions, and for migraine, whether interventions are for acute or preventive treatment.

The cost-effectiveness threshold – the maximum amount willingly paid for a unit of health outcome – may depend on gross domestic product per capita (80). If the estimated cost-effectiveness of a new technology compared with a suitable alternative falls below the threshold, the new technology will likely be recommended. However, for values near the threshold, the level of uncertainty may become important. Thresholds are often established by analysis of previous (reimbursement) decisions; they are not themselves outputs of cost-effectiveness analyses, but guides (or rules) to interpretation of these outputs for decision-making, and they are specific to each unit of health outcome used. Thresholds are closely related to the economic concept of opportunity cost, wherein the value of a technology is equivalent to the value of what was foregone in order to use it. The threshold value stands for the health outcome that could have been achieved if the resource required to implement the intervention of interest had been used elsewhere. Although some countries (e.g. the National Institute for Health and Care Excellence for England and Wales) make the thresholds that they use explicit, others do not and some vary them by healthcare sector or disease area.

## Public health impact

In keeping with the goal of providing policymakers with a comprehensive, evidence-based review, it is essential that HTAs incorporate data about the public health impact of technologies applied in patients with migraine (medications and/or neuromodulatory devices). Assessments should communicate the potential impact of new migraine treatments in specific healthcare settings and geographic locations, as well as on the families or partners of affected patients, while taking into account the size of the population and the prevalence of the disease. Additional elements of HTAs should examine migraine’s impact on healthcare systems and communities and consider the potential benefits of professional training and extending access to care for individuals in high-risk populations.

## Alignment with IHS clinical trial guidelines

To ensure that HTA recommendations align with IHS clinical trial guidelines for migraine, this position statement will be revised whenever a new edition of a guideline published by the IHS supersedes it.

## Methodology used for the development of these guidelines

The IHS formed a Committee and assigned it the task of developing a position statement on HTAs for the acute and preventive treatment of migraine. Funding for editorial assistance was provided by the IHS. The Committee’s work was independent and unbiased, and the process of developing this statement involved two phases. The initial draft of the statement was written by the first author and individual members of the committee based on HTA recommendations from the European Union (28), the French National Authority for Health (*Haute Autorite de Sante*) (81), the National Institute for Health and Care Excellence, for England and Wales Institute for Clinical and Economic Review. A revision incorporating the collaborative work of all Committee members was sent to pharmaceutical manufacturers and patient associations for feedback. The resulting draft was discussed at a face-to-face meeting in Copenhagen in February 2020 and a videoconference in July 2020. After incorporating the views of all stakeholders, the position statement was approved by the Board of the International Headache Society.

## Public health relevance

- The Clinical Trials Subcommittee of the International Headache Society presents the first Health Technology Assessment for the Acute Treatment of Migraine Attacks and Prevention of Migraine: A Position Statement of the International Headache Society.
- The objectives of this position statement are to recommend global standards for the collection and analyses of evidence pertaining to new technologies (medications and devices) for the treatment of migraine and to facilitate HTAs that account for the distinctive nature of migraine and the heterogeneity of the affected population.
